# Antibacterial activity and cytotoxicity of a novel bacteriocin isolated from *Pseudomonas* sp. strain 166

**DOI:** 10.1111/1751-7915.14096

**Published:** 2022-07-18

**Authors:** Yu Wang, M. Aman Haqmal, Yue‐dong Liang, Inam Muhammad, Xiao‐Ou Zhao, Emad Mohammed Elken, Yun‐Hang Gao, Yu Jia, Cheng‐guang He, Yi‐Ming Wang, Ling‐Cong Kong, Hong‐Xia Ma

**Affiliations:** ^1^ College of Veterinary Medicine Jilin Agricultural University 130118 Changchun, Jilin China; ^2^ Department of Animal Sciences Shaheed Benazir Bhutto University Sheringal Dir Upper‐Pakistan Sheringal Pakistan; ^3^ Animal Production Department Faculty of Agriculture Al‐Azhar University Nasr City, Cairo 11884 Egypt; ^4^ Jilin Agricultural University College of Life Science Changchun China; ^5^ The Key Laboratory of New Veterinary Drug Research and Development of Jilin Province Jilin Agricultural University Xincheng Street No. 2888 Changchun 130118 China; ^6^ The Engineering Research Center of Bioreactor and Drug Development, Ministry of Education Jilin Agricultural University Changchun China

## Abstract

*Pseudomonas* sp. strain 166 was isolated from soil samples from Changbai Mountains. A novel bacteriocin PA166 from *Pseudomonas* sp. 166 was purified using ammonium sulfate, dextran gel chromatography column and Q‐Sepharose column chromatography successively. The molecular mass of bacteriocin PA166 was found to be 49.38 kDa by SDS‐PAGE and liquid chromatography–mass spectrometry (MS)/MS. Bacteriocin PA166 showed stability at a wide range of pH (2–10), and thermal stability (40, 60, 80 and 100°C). The bacteriocin PA166 antimicrobial activity was slightly inhibited by Ca^2+^, K^+^ and Mg^2+^. The minimum bactericidal concentrations of bacteriocin PA166 against five *Pasteurella multocida* strains ranged from 2 to 8 μg ml^−1^. Bacteriocin PA166 showed low cytotoxicity and a higher treatment index (TI = 82.51). Fluorescence spectroscopy indicated that bacteriocin PA166 destroyed the cell membrane to exert antimicrobial activity. In summary, bacteriocin PA166 had strong antibacterial activity, high TI and low toxicity, and hence could serve as a potential clinical therapeutic drug.

## Abstract 2

Bacteriocin PA166 had strong antibacterial activity, high TI, and low toxicity, and hence could serve as a potential clinical therapeutic drug.

## Introduction

Bovine respiratory disease (BRD) is one of the most common diseases in the beef cattle breeding sector. Its major pathogens have resulted in significant drug resistance to routinely used clinical medications. According to statistics, anti‐infective failure is estimated to cost between 800 and 900 million dollars each year. Therefore, new antimicrobial drugs for treating primary BRD infections are urgently needed. *Pasteurella multocida* is one of the BRD pathogens, and recently extensively drug‐resistant strains have become prevalent (Snyder and Credille, 2020). Therefore, new antimicrobial drugs for treating primary *P. multocida* infections are urgently needed. Bacteriocins are considered substitutes for antibiotics and have been of widespread concern.

Bacteriocins are proteins or short peptides with antibacterial activity, which are synthesized by ribosomes of different kinds of bacteria. Previous studies suggested that bacteriocins acted only with bacteria of the same species or related species. However, in recent years, bacteriocins refer to an antibacterial peptides with activity against various microorganisms, which are not closely related but have a specific immune mechanism of the producer themselves (Cotter *et al*., 2013). Bacteriocins can inhibit the growth of bacteria, fungi and viruses (Hernández‐González *et al*., 2021), and hence have drawn substantial attention. Many antibiotics are derived from special metabolites produced by bacteria (Culp *et al*., 2020). Bacteriocins have various modes of functions, including membrane permeability, cell wall damage, blocking of metabolic pathways (Hols *et al*., 2019) and pore formation (Kumariya *et al*., 2019), reducing the risk of cross‐resistance development (Soltani *et al*., 2021). Bacteriocins combined with other antimicrobial agents or antibiotics with different mechanisms can improve their antibacterial activity and reduce the risk of drug resistance (Soltani *et al*., 2021).

Bacteriocins showed minimal cytotoxicity to host cells (Baindara *et al*., 2018). The mice were given 0.5 mg kg^−1^ bacteriocin TSU4 daily for 21 days without death or treatment‐induced changes in their physiological status, indicating that the bacteriocin was nontoxic (Sahoo *et al*., 2017). Marlida et al. showed that the lactocin 160 solution was injected into female rabbits through the vagina; it had no serious irritating effect on the vaginal epithelium or no toxicity to lactobacilli (Dover *et al*., 2007). OG716 was a derivative of mutacin1140 and did not show any side effects when taken orally by Golden Syrian hamsters (Pulse *et al*., 2019). Bacteriocins may be safe, as shown by a few studies reported so far.

*Pseudomonas* is a diverse genus with more than 60 species that exhibit different lifestyles in a variety of environments. They are ubiquitous in nature, producing a large number of secondary metabolites (Gross and Loper, 2009). R‐type pyocins produced by *Pseudomonas aeruginosa* have potential as therapeutics in infection (Mei *et al*., 2021). The bacteriocin‐like substance could be against other *Pseudomonas* strains and food‐borne pathogens (Ghrairi *et al*., 2015). Pyocin 611131 shows bactericidal activity against *Neisseria gonorrhoeae* and other species of Neisseria (Morse *et al*., 1976). Bacteriocin PA996 isolated from *Pseudomonas azotoformans* showed antibacterial activity only against *P. multocida* (Wang *et al*., 2022). This study explored the purification of bacteriocin PA166 from *Pseudomonas* sp. strain 166 and then its characterization, aiming to find a safe substitute for antibiotics with strong antibacterial activity.

## Results

### Isolation and identification of the bacteria with antibacterial activity

**Fig. 1:**
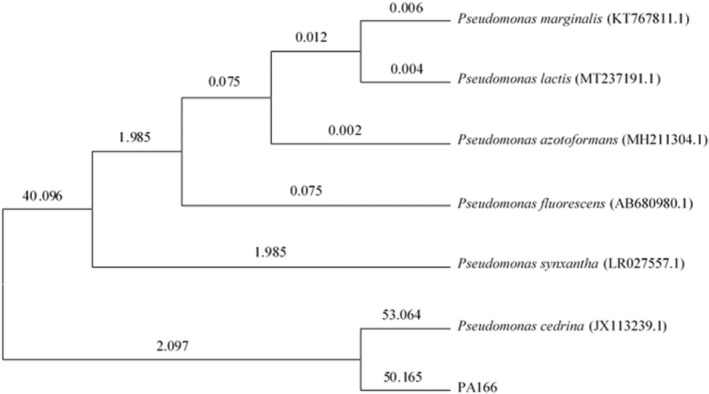
The phylogenetic tree of 16S rDNA gene sequences for isolated strain. The bootstrap values (%) are shown at the branches from 1000 replications.

The nucleotide sequence of the partial 16S rDNA gene sequence of *Pseudomonas* sp. strain 166 was shown in Appendix S1. To further determine strain 166, a phylogenetic tree was generated to compare its 16S rDNA to that of other *Pseudomonas* sp. As shown in Fig. 1, a phylogenetic tree was constructed with other trains of *Pseudomonas* sp. The gene sequence showed 98% homology with *Pseudomonas cedrina* strain Y310 (GenBank number JX113239.1), indicating that the strain could be a novel species and should be named *Pseudomonas* sp. strain 166.

### Purification of bacteriocin PA166 from *Pseudomonas* sp*.*

In this study, the crude bacteriocin PA166 was purified in three steps. The crude extract was loaded on a dextran gel column. Then, the active fractions were purified using Q‐Sepharose column chromatography. The binding protein was further purified by stepwise elution and linear gradient elution. The enzyme was purified using ion‐exchange chromatography involving two types of elution: stepwise and linear‐gradient (Chafik *et al*., 2020). The results of the purification process results are shown in Fig. S1.

### Molecular mass determination of bacteriocin PA166

**Fig. 2:**
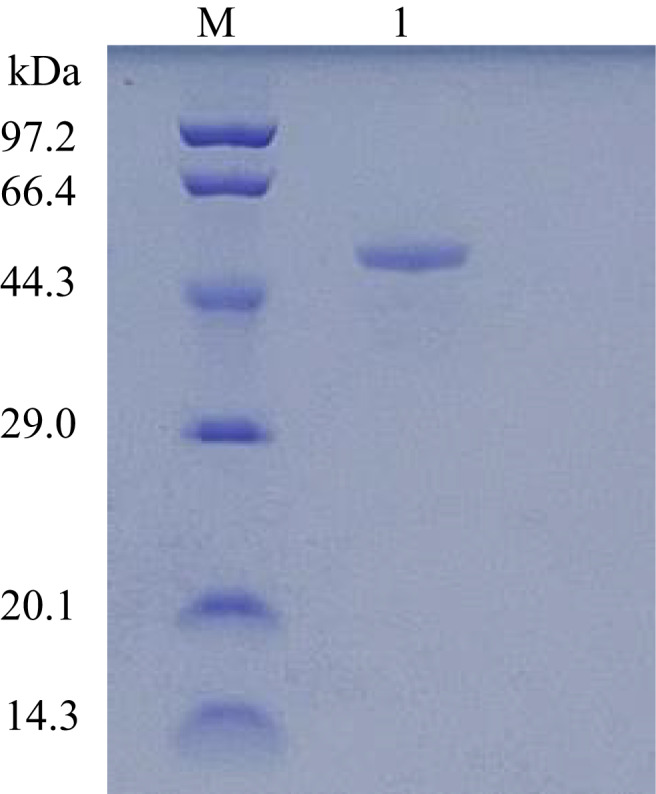
Molecular mass determination of bacteriocin PA166. M: low molecular marker; 1: bacteriocin PA166 loaded on the gel.

SDS‐PAGE showed a protein band between approximately 44 and 66 kDa (Fig. 2). The liquid chromatography–MS/MS result showed a fragment of mass 49.38 kDa (Fig. S2).

### Stability of bacteriocin PA166

The effects of temperature, pH, cations (Na^+^, K^+^, Ga^2+^ and Mg^2+^), and different chemicals were investigated. The results summarized in Table 1 showed that different enzymes, temperatures, pH, and Na^+^ did not affect the antimicrobial activity of bacteriocin PA166. However, the antimicrobial activity was inhibited by Ca^2+^, K^+^, Mg^2+^, EDTA, Triton X‐100, β‐ME, Tween 20 and Tween 80. The relative activity was 85.18%, 92.60%, 85.18%, 60%, 60%, 80%, 75% and 90% respectively.

**Table 1:** Effect of different treatments on the activity of bacteriocin PA166.

Conditions	Relative activity(%)
Control	100 ± 0.02
Temperature (°C)	
40	100 ± 0.015
60	100 ± 0.036
80	100 ± 0.023
100	100 ± 0.020
Enzyme	
Proteinase K	0
Catalase	100 ± 0.0156
Trypsin	100 ± 0.018
Papainc	100 ± 0.022
pH	
2	100 ± 0.041
3	100 ± 0.029
4	100 ± 0.012
5	100 ± 0.024
6	100 ± 0.012
7	100 ± 0.033
8	100 ± 0.050
9	100 ± 0.045
10	100 ± 0.032
Cations	
Na^+^	100 ± 0.012
K^+^	85.18 ± 0.021
Ga^2+^	92.60 ± 0.011
Mg^2+^	85.18 ± 0.033
Chemicals	
PMSF	100 ± 0.0136
Cystein	100 ± 0.028
β‐ME	80 ± 0.053
DTT	100 ± 0.463
EDTA	60 ± 0.017
Triton X‐100	80 ± 0.086
Tween −20	75 ± 0.031
Tween −80	90 ± 0.029
Ethanol	100 + 0.0373

### Antibacterial spectrum of bacteriocin PA166

Bacteriocin PA166 was found to be active against *P. multocida*, *M. haemolytica*, *E. faecium* and MRSA, and had an excellent inhibitory effect on *P. multocida*. The result was shown in Table 2.

**Table 2:** Antibacterial spectrum of bacteriocin PA166.

Indicator organisms	Zone of inhibitions (mm)
Gram‐negative bacteria	
*P. multocida* ATCC 43137	40
*P. multocida* 32	38
*P. multocida* 6	45
*P. multocida* 16	35
*P. multocida* 21	41
*E. coli* ATCC 25922	0
*E. coli* B2	0
*E. fergusonii*	0
*M. haemolytica*	32
*S. enterica* subsp. enterica ATCC H9812	0
*P. Aeruginosa*	0
Gram‐positive bacteria	
*E. faecalis*	0
*E. faecium*	22
*S. gallinarum*	25
*T*. pyogenes	0
MRSA	23
Fungus	
*C. albicans*	0
*Phaffia rhadozyma*	0

### Antimicrobial activity

**Fig. 3:**
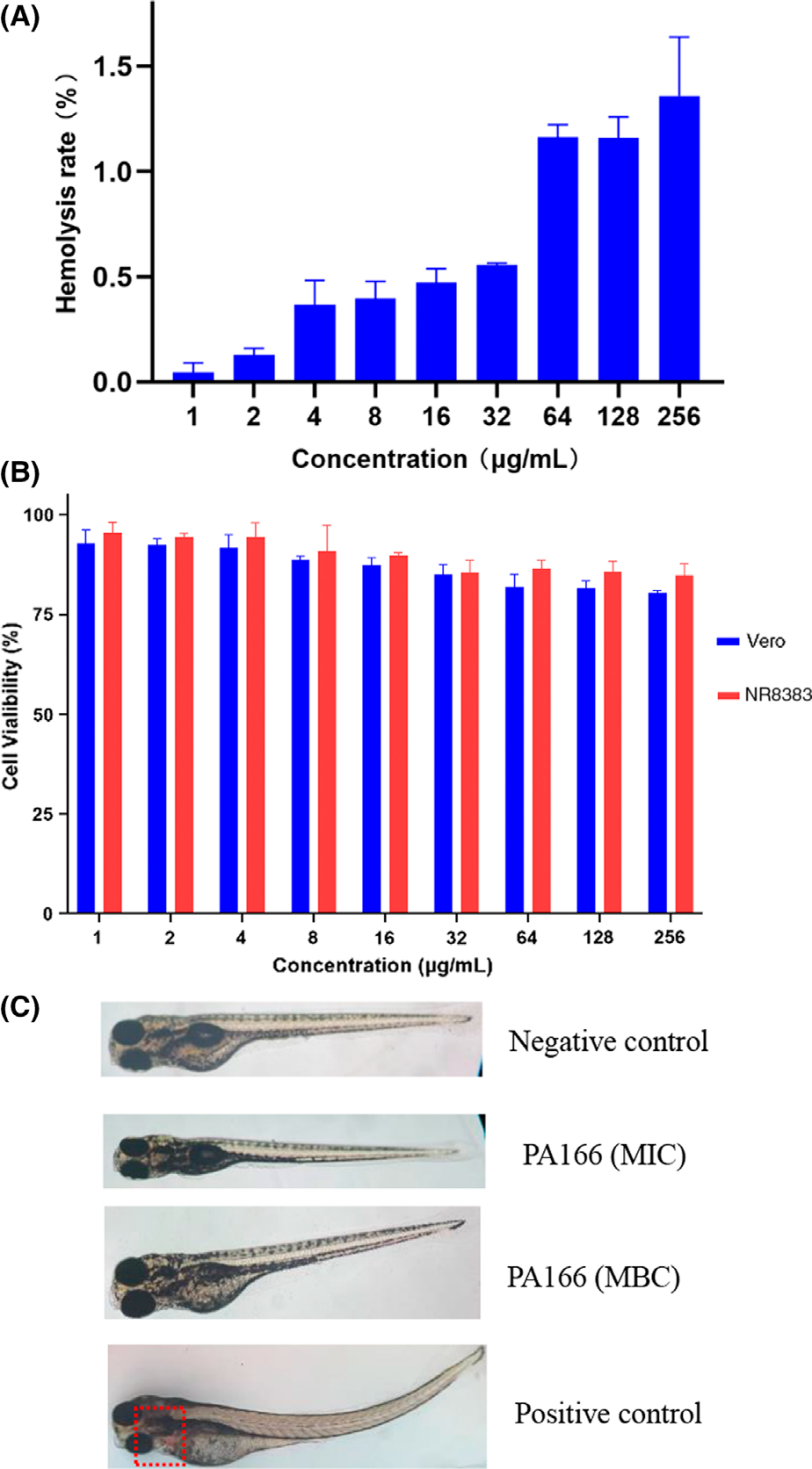
Safety analysis of bacteriocin PA116. A. Hemolytic activity of bacteriocin PA116 against rabbit red blood cells, (B) cytotoxicity of the bacteriocin PA116 against Vero cells (blue) and NR8383 cells (red) and (C) zebrafish embryos were either treated with embryo medium as a negative control,bacteriocin PA166 (MIC, MBC) or sodium dehydroacetate (200 μg ml^−1^) as a positive control.

Table 3 shows the MBCs of bacteriocin PA166 against five *P. multocida* strains; the MBCs ranged from 2 to 8 μg ml^−1^. The treatment index (TI) is the safety index of drugs. The geometric mean was calculated using MHC and MBC (Wang *et al*., 2015). The larger the value of the therapeutic index, the greater the antimicrobial specificity. When no hemolytic activity was detected using 250 μg ml^−1^, the TI was calculated using 500 μg ml^−1^ (Chen *et al*., 2005). Figure 3A shows the hemolytic activity at bacteriocin PA166 concentrations ranging from 1 to 256 μg ml^−1^. The bacteriocin had a low hemolysis rate, which was only 1.36% in 256 μg ml^−1^.

**Table 3:** Effect of bacteriocin PA166 on the biological activity.

Indicator organisms	MIC (μg ml^−1^)	MBC (μg ml^−1^)	Therapeutic index
*P. multocida* ATCC 43137	2	8	82.51
*P. multocida* 32	1	4	
*P. multocida* 6	2	4	
*P. multocida* 16	1	2	
*P. multocida* 21	1	2	

### Cytotoxicity measurement

The cytotoxicity of bacteriocin PA166 was evaluated using Vero and NR8383 cells. Figure 3B shows cell viability. The hemolysis rate increased with the increase in bacteriocin PA166 concentration, but Vero and NR8383 cells still remained 80% viable at a concentration of 256 μg ml^−1^.

### Zebrafish embryo developmental toxicology assays

As illustrated in Fig. 3C, the positive control showed the typical phenotype of epicardial haemorrhage. Compared with the control, no acute toxic and teratogenic effects were observed in zebrafish at different concentrations of the bacteriocin PA166.

### Mouse infection model

**Fig. 4:**
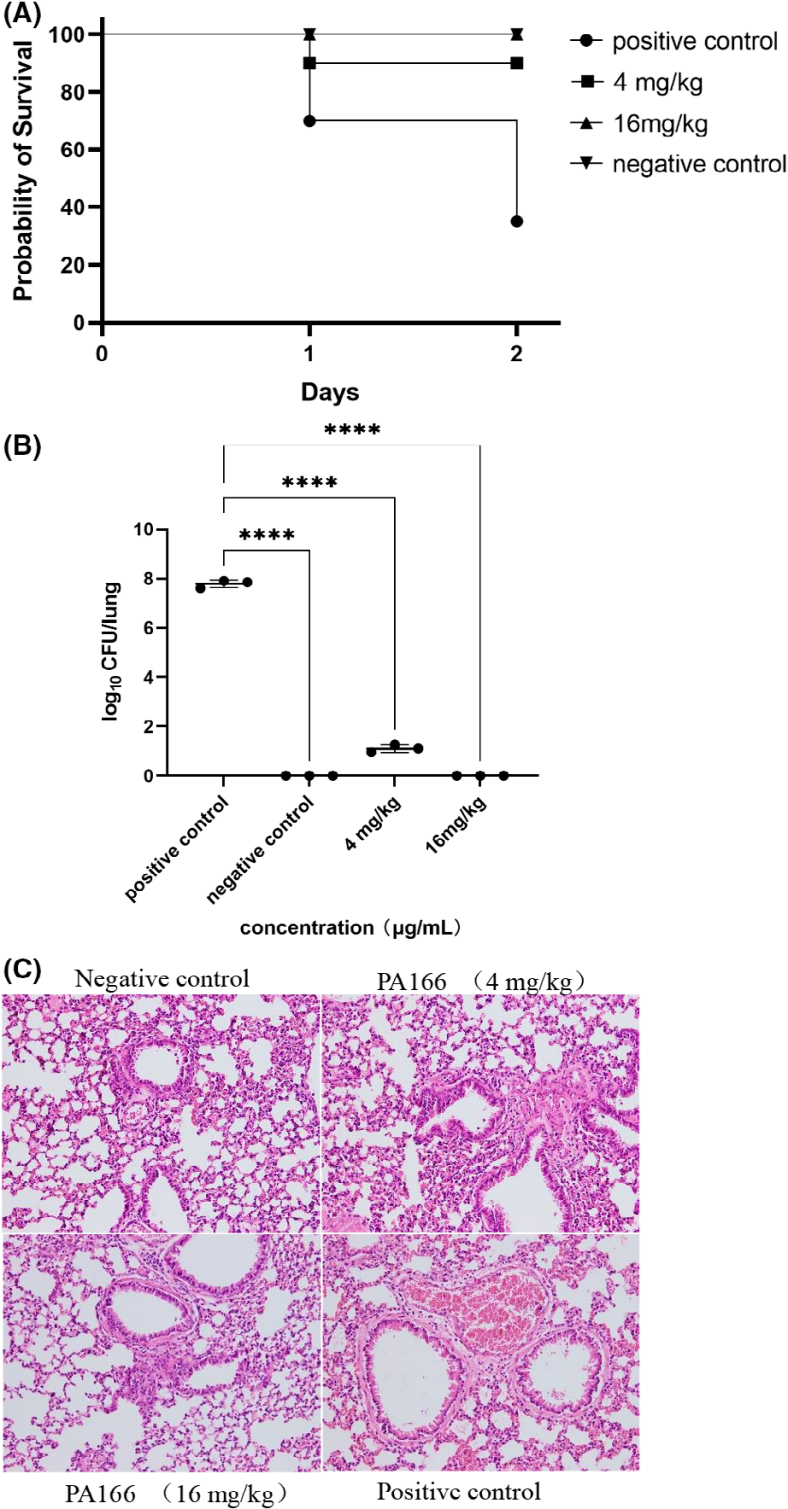
Therapy of *P. multocid* infection in mice (A) survival rates of mice (*n* = 10), PA166 treatment of lethal dose of *P. multocida* (1.12 × 10^6^ CFU ml^−1^) improves the survival rate of mice. B. The amount of bacteria in the lungs of mice treated with antimicrobial substances (****P < 0.0001.) and (C) lung tissues with H&E staining (200x).

The therapeutic effect of bacteriocin PA166 was further evaluated in an animal model infected with *P. multocida*. On day 2 after infection, the survival rate of mice treated with bacteriocin increased significantly. The survival rate of mice was more than 90% at a bacteriocin concentration of 4 mg kg^−1^ and reached 100% at the bacteriocin concentration of 16 mg kg^−1^ (Fig. 4A). We quantified the number of bacteria in the lungs to evaluate germicidal efficacy (Fig. 4B). Obviously, the PA166 treatment significantly reduced the number of bacteria, indicating that PA166 had strong antibacterial effects.

*Pasteurella multocida* is the predominant pathogen that causes bovine pneumonia. Moreover, the changes in the lung tissue were evaluated using H&E staining. As illustrated in Fig. 4C, the 4 mg kg^−1^ bacteriocin PA166 caused local thickening of the pulmonary interstitium, exfoliated cell fragments in part of the alveolar cavity, and complete tracheal cilia structure. However, the control and 16 mg kg^−1^ bacteriocin PA166 treatment groups showed that the alveolar structure was normal, the alveolar cavity was clean, the alveolar wall was not thickened and the trachea cilia were intact. The positive group presented blood cells in the alveolar cavity, broken tracheal cilia and dilated alveolar cavity.

### Time‐killing kinetics

**Fig. 5:**
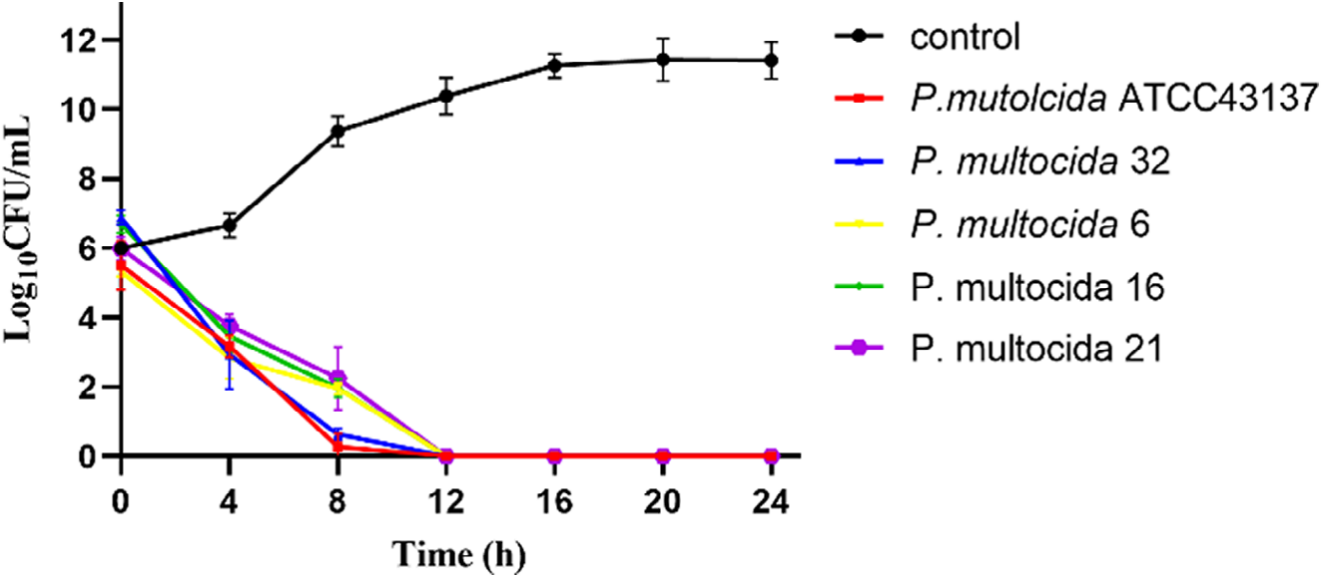
Time‐killing curves of bacteriocin PA166 on different *P. multocida*.

As shown in Fig. 5, the ability to kill different *P. multocida* strains with PA166 (the concentrations of MBC) was determined. At MBC, all the bacteria were killed by PA166 after 12 h.

### Release of ATP and ROS

**Fig. 6:**
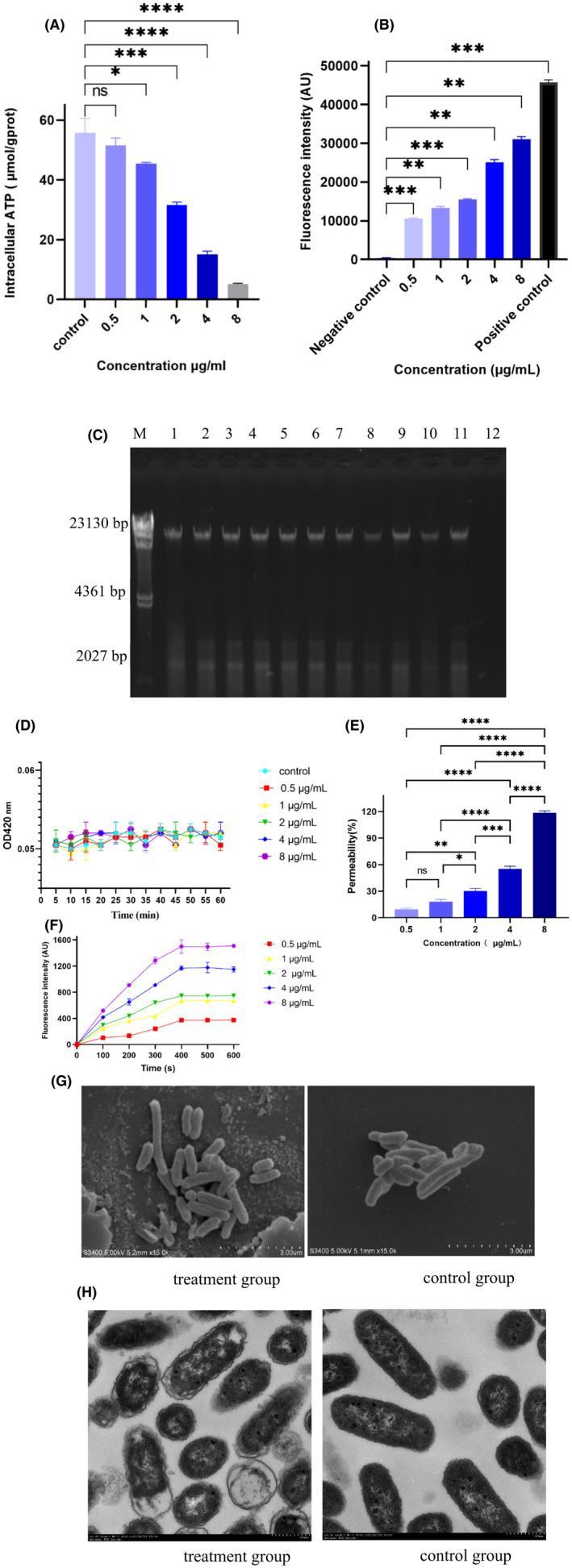
Mechanism of PA116. A. Decreased levels of intracellular ATP in *P. multocida* ATCC43137 by treatment with PA116. B. Total ROS accumulation in *P. multocida* ATCC43137 treatment using PA116. C. A gel retardation experiment was used to measure the DNA binding assay. M: λ/HindIII DNA Marker; 1: genomic DNA alone; 2–11: 0.5–256 μg ml^−1^ PA116; 12: 256 μg ml^−1^ PA116 alone. D. Inner membrane permeability of *P. multocida* after the treatment of PA116. E. Outer membrane permeability of *P. multocida* ATCC43137 treatment using PA116. F. Cytoplasmic membrane depolarization of *P. multocida* ATCC43137 by the PA116; g SEM images of *P. multocida*. H. TEM images of *P. multocida*. *P < 0.05, **P < 0.01, ***P < 0.001 and ****P < 0.001.

As shown in Fig. 6A, bacteriocin PA166 decreased intracellular ATP in a dose‐dependent manner in *P. multocida* ATCC43137. Moreover, bacteriocin PA166 induced the accumulation of ROS (Fig. 6B), which aggravated the damage to the membrane and further destroyed the homeostasis of bacteria (Song *et al*., 2020).

### DNA‐binding assay

DNA‐binding properties of bacteriocin PA166 were examined. As shown in Fig. 6C, bacteriocin PA166 did not inhibit DNA migration at different concentrations.

### Inner membrane permeability

The cytoplasmic membrane permeability was tested by determining the release of cytoplasmic β‐galactosidase. As illustrated in Fig. 6D, bacteriocin PA166 could not change the permeability of the cytoplasmic membrane.

### Outer membrane permeability assay

The hydrophobic fluorescence probe NPN could evaluate the outer membrane permeability. NPN was excreted from the outer membrane and quenched in the aqueous environment, but when the outer membrane was disturbed, NPN entered the cell, which caused fluorescence enhancement under hydrophobic conditions (Wang *et al*., 2019). As illustrated in Fig. 6E, bacteriocin PA166 showed dose dependence on the outer membrane, indicating that bacteriocin PA166 was permeable to the outer membrane of *P. multocida*. Above all, these studies showed that bacteriocin PA166 could kill bacteria by penetrating the outer membrane of *P. multocida* ATCC 43137.

### Cytoplasmic membrane depolarization

The membrane potential–sensitive dye DiSC3(5) could be used to determine the ability of peptides to change cytoplasmic membrane potential (Zhu *et al*., 2014). As shown in Fig. 6F, bacteriocin PA166 could permeate the bacterial cell membrane in a dose‐dependent manner. When the cell membrane permeated, the membrane potential dissipated and DiSC3(5) was released, leading to the enhancement of fluorescence (Lv *et al*., 2016).

### SEM and TEM

The bacterial membrane damage of *P. multocida* ATCC 43137 treated with bacteriocin PA166 was detected using SEM. Compared with the control cells, the cell membrane of the treated cells was dramatically damaged (Fig. 6G). TEM was used to investigate the interaction of *P. multocida* ATCC 43137 treated with bacteriocin PA166 so as to visually observe the intracellular ultrastructural alterations. The control cells had a bright and smooth surface, and the bacterial cell membrane was significantly ruptured (Fig. 6H).

## Discussion

The soil microbiome is complex and important. It is an integral part of the terrestrial ecosystem because it contains a large number of microorganisms, including bacteria, fungi, archaea, viruses and protozoa (Islam *et al*., 2020). Soil bacteria are the main source of special antibacterial metabolites (Pahalagedara *et al*., 2021). *Pseudomonas* exists in various environments such as soil, water, plant surfaces and animals (Michel‐Briand and Baysse, 2002). Plant lectin‐like bacteriocin isolates *Pseudomonas* sp. strain BW11M1,which kills *Pseudomonas putida* GR12‐2R3 (Parret *et al*., 2003). A novel *P. aeruginosa* puBac bacteriocin has been shown to have significant effects on *E. coli*, *Staphylococcus* sp. and *Bacillus* sp. (Lakshmanan *et al*., 2020). S‐type pyocins produced by *P. aeruginosa* could kill other strains of the same species (Dingemans *et al*., 2016). In this study, *Pseudomonas* sp. 166 was isolated from soil, which could produce bacteriocin PA166 with bacteriostatic activity. The gene sequence of *Pseudomonas* sp. strain 166 showed 98% homology with *P. cedrina* strain Y310, indicating that the strain was a novel species.

Due to the abuse of antibiotics, drug resistance has become more serious, and attention has been paid to the research and development of new drugs. Bacteriocins are a promising substitute in the research of alternative antibiotics (Guo *et al*., 2020). They have a wide range of applications, including food industry (Gálvez *et al*., 2007; Alvarez‐Sieiro *et al*., 2016), clinical applications (Rea *et al*., 2011), agriculture applications (Yu *et al*., 2019) and veterinary medicine (van Heel *et al*., 2011; Yu *et al*., 2019; Naimi *et al*., 2020). They exert a strong inhibitory effect on a variety of (multidrug‐resistant) bacteria; the tendency to develop drug resistance is also low (van Heel *et al*., 2011). Bacteriocin PA166 was purified using ammonium sulfate, dextran gel column chromatography and Q‐Sepharose column chromatography. A single protein band with a molecular weight of 49.38 kDa was obtained. Bacteriocin PA166 work against *P. multocida*, *M. haemolytica*, *Salmonella gallinarum*, *E. faecium*, and MRSA. The stability of bacteriocins is a standard to evaluate the application of bacteriocins (Qiao *et al*., 2020a, 2020b). Bacteriocin PA166 was stable for different temperatures, which was similar to other bacteriocins, such as Enterocin TJUQ (Qiao *et al*., 2020a, 2020b). Bacteriocin PA166 was active at pH 2–12, which suggested that bacteriocin PA166 was resistant to alkaline and acidic conditions. Effect of various cations, Na^+^, Ca^2+^, K^+^ and Mg^2+^ on the activity of bacteriocin PA166, the antimicrobial activity was slightly inhibited by Ca^2+^, K^+^ and Mg^2+^. The antimicrobial activity was also slightly inhibited by EDTA, Triton X‐100, β‐ME, Tween 20 and Tween 80. But its antibacterial activity disappears completely under the action of proteinase K, which indicated the proteinaceous character of bacteriocin PA166. However, soluble or S‐type pyocins from *P. aeruginosa* were protease‐ and heat‐sensitive bacteriocins (Ghequire and De Mot, 2014). Bacteriocin PA996 isolated from *P. azotoformans* was only against *P. multocida*, which showed antibacterial activity in the range of pH2‐10, but it was heat‐labile (Wang *et al*., 2022).

The bactericidal mechanisms of bacteriocins include permeabilization of the cytoplasmic membrane, dissipation of the proton motive force, inhibition of protein DNA, RNA and peptidoglycan synthesis and cell lysis (Montville and Bruno, 1994). The common antibacterial mechanism of bacteriocin that destroys the cell membrane is as follows. Bacteriocin binds to a specific receptor protein on the membrane, thereby forming a pore, which increases the permeability of the membrane and leads to cell death (Ríos Colombo *et al*., 2019; Cui *et al*., 2021). Bacteriocin PA166 showed significant antimicrobial activity against five *P. multocida* strains, the MBCs ranging from 2 to 8 μg ml^−1^. After PA166 treatment, the death of mice and the number of bacteria in the lung were significantly reduced, suggesting that PA166 was an effective antimicrobial therapy. The bacteriocin PA166 did not attach to the DNA of *P.multocida* ATCC43137 in the range of 0.5–256 μg ml^−1^, suggesting bacteriocin PA166 does not exert its antibacterial activity by binding to the DNA of *P. multocida*. SEM, TEM and outer membrane permeability assay showed that bacteriocin PA166 could destroy the integrity and increase the permeability of the cell membrane. The destruction of cell membrane integrity caused by bacteriocin PA166 was further determined by the release of ATP and ROS. Bacteriocin induces cell membrane permeability and intracellular ATP depletion of target bacteria (Christensen and Hutkins, 1992). *Pseudomonas aeruginosa* PAO1 encodes a novel S‐type pyocin whose antimicrobial mechanism is membrane damage, manifested by intracellular substance leakage, increased membrane permeability, and cell surface disruption (Ling *et al*., 2010). The changes in the integrity of the cell membrane lead to the complete disintegration of the cell (McGrath *et al*., 2013; Lu *et al*., 2020). Hence, the results showed that Bacteriocin PA166 mediated membrane‐disruptive mechanism. These results were similar to a short linear antibacterial peptide (SLAP)‐S25 (Song *et al*., 2020) and bacaucin (Liu *et al*., 2017).

Most studies evaluated the safety of bacteriocins by determining hemolytic activity (Qiao *et al*., 2020a, 2020b). Although PA166 had excellent antibacterial properties and good stability, its biosafety was also worthy of further consideration. In this study, the biosafety of PA166 was evaluated using cytotoxicity measurement therapeutic index and zebrafish embryo developmental toxicology assays. PA166 showed less cytotoxicity at higher concentrations and had a higher treatment index. Moreover, PA166 had no acute toxicity and teratogenic effect on zebrafish.

## Conclusions

In conclusion, bacteriocin PA166 was successfully purified, which exhibited significant antimicrobial activity against *P. multocida*. Bacteriocin PA166 killed indicator bacteria mainly by destroying the out membrane. Bacteriocin PA166 had good safety, therapeutic effect and physicochemical stability. New antibiotics are urgently needed to treat infections caused by antibiotic‐resistant bacteria. Therefore, bacteriocin PA166 has the potential to serve as an effective and safe antibacterial agent.

## Experimental procedures

### Materials

Mueller‐Hinton agar (MH), MH broth, Lysogeny Broth (LB) broth and DNA rapid extraction kit were purchased from Sangon Biotech (Shanghai, China) Co., Ltd., (NH_4_)_2_SO_4_ was purchased from Beijing Dingguo ChangSheng Biotechnology Co., Ltd (Beijing, China). The cutoff membrane, cell counting kit‐8 assay (CCK8 assay), proteinase K, catalase, trypsin and papain were purchased from Beijing Solarbio Science & Technology Co., Ltd (Beijing, China). ATP assay and reactive oxygen species (ROS) assay kits were purchased from Nanjing Jiancheng Bioengineering Institute (Nanjing, China). Further, 2‐nitrophenyl β‐d‐galactopyranoside (ONPG) was purchased from Shanghai Macklin Biochemical Co., Ltd., and *N*‐phenyl‐1‐naphthylamine (NPN) was purchased from Sigma–Aldrich (Shanghai, China).

### Bacterial strains and growth conditions

*Pasteurella multocida* ATCC 43137 (*P. multocida* ATCC43137), *P. multocida* 32 (quinolone‐resistant strain), *P. multocida* 6 (tetracycline‐resistant strain), *P. multocida* 16 (erythromycin‐resistant strain), *P. multocida* 21 (spectinomycin‐resistant strain), *Escherichia coli* ATCC 25922 (*E. coli* ATCC 25922), *E. coli* B2 (multidrug‐resistant bacteria), *Escherichia fergusonii* (*E. fergusonii*), *Mannheimia haemolytica* (*M. haemolytica*), *Salmonella enterica* subsp. enterica ATCC H9812 (*S. enterica* subsp. enterica ATCC H9812)*, Pseudomonas aeruginosa* (*P. aeruginosa*), *Enterococcus faecalis* (*E. faecalis*), *Enterococcus faecium* (*E. faecium*), methicillin‐resistant *Staphylococcus aureus* (MRSA), *Trueperella pyogenes* (*T. pyogenes*), *Phaffia rhodozyma* and *Candida albicans* (*C. albicans*) were used as indicator organisms. The culture conditions for all indicator bacteria are listed in Table S1.

### Strain isolation

According to a previous study (Gastauer *et al*., 2015), bacteriocin‐producing strains were isolated from soil samples from Changbai Mountains in the Jilin province, China. Briefly, each altitude (< 1000, 1000–1500, 1500–1700, 1700–2000 and > 2000 m) is divided into three sampling sections, the soil samples in each location are indicated as 18 sampling points in a 4 × 300 m sampling square. A soil sampler collects topsoil within 15 cm of each sampling point, and the samples from 18 sampling sites are evenly mixed to remove gravel, plant waste, and other contaminants before being kept in aseptic bags. Isolation of antagonistic *P. multocida* strains, the strain with the highest inhibitory activity was selected for further study.

The strain was identified according to a previous study (Dong *et al*., 2019a, 2019b), specific primers were 27F (5′‐AGAGTTTGATCCTGGCTCAG‐3′), 1492R (5′‐GGTTACCTTGTTACGACTT‐3′). PCR parameters were 94°C for 5 min, 28 cycles of 94°C for 30 s, 55°C for 45 s and 72°C for 90 s, and a final extension at 72°C for 5 min. The polymerase chain reaction products were sent to Sangon Biotech (Shanghai) Co., Ltd. for sequencing.

### Purification of bacteriocin PA166

#### Preparation of crude bacteriocins

Three microliters of *Pseudomonas* sp. strain 166 (OD 0.5 at 600 nm) were inoculated in 300 mL of LB broth and cultured at 16°C and 100 rpm for 48 h (OD 2.42 at 600 nm). The cells were removed by centrifugation (8000 *g* for 30 min at 4°C). Saturated 80% ammonium sulfate was added to the supernatant, stirred for 6 h, and allowed to stand overnight at 4°C. Subsequently, PBS (pH 6) was added to dissolve the precipitate. The crude bacteriocins were dialyzed at 4°C with a 5‐kDa dialysis bag (Qiao *et al*., 2020a, 2020b).

#### Purification by dextran gel column

Crude bacteriocins from *Pseudomonas* sp. strain 166 were loaded onto a dextran gel column (16 × 1000 mm, G75) balanced with PBS. The flow rate was 0.3 ml min^−1^, and 3‐ml fractions were collected. The antibacterial activity of bacteriocin PA166 against indicator bacteria was detected using the agar well diffusion assay (Fredua‐Agyeman and Gaisford, 2019). Briefly, 10^6^ CFU ml^−1^ of *P. multocida* was spread onto MH agar plates (containing 5% serum), wells were made with a sterile Oxford cup and filled with 100 μl of the collected fractions. Then the agar plates were kept for 3 h for the diffusion of the fractions from the wells into the agar and incubated at 37°C for 16 h.

#### Purification by Q‐Sepharose column

Bacteriocin PA166 was further purified as previously described (Chafik *et al*., 2020). The concentrated antibacterial fraction was loaded onto a Q‐Sepharose column. Bacteriocin PA166 was eluted with different concentrations of NaCl prepared in PBS. The flow rate was 1 ml min^−1^, and 3‐mL fractions were collected. The antibacterial activity of bacteriocin PA166 against indicator bacteria was detected using an agar well diffusion assay (Fredua‐Agyeman and Gaisford, 2019). Then, bacteriocin PA166 with antibacterial activity was dialyzed at 4°C using a 5‐kDa cutoff membrane (Qiao *et al*., 2020a, 2020b).

The antibacterial fraction was loaded onto a Q‐Sepharose column. Bacteriocin PA166 was eluted using a linear gradient of NaCl (0–500 mM) prepared in PBS. The flow rate was 1 ml min^−1^. Bacteriocin PA166 was concentrated and stored at −80°C for further study.

### Molecular mass determination

The mass of bacteriocin PA166 was determined using SDS‐PAGE (12% gel). The gel was run at 100 V for 30 min and then at 200 V for 1 h. Then gel was stained using the Coomassie brilliant blue staining method.

Further, the mass of bacteriocin PA166 was also determined using MALDI‐TOF mass spectrometry (MS). Briefly, the band was cut and sent to Sangon Biotech (Shanghai) Co., Ltd. for determining the molecular mass of bacteriocin PA166.

### Stability of bacteriocin PA166

Bacteriocin PA166 was treated with trypsin, proteinase K, papain and catalase (final concentration of 1 mg ml^−1^) at 37°C for 1 h. The untreated bacteriocin PA166 was used as control.

Bacteriocin PA166 was incubated at 60, 80 and 100°C for 30 min to test the effect of temperature on bacteriocin PA166 activity. The untreated bacteriocin PA166 was used as control.

The pH (from 2 to 10) of bacteriocin PA166 was adjusted using HCl and 1 M NaOH solution; after 1 h it was then adjusted back to 7. The untreated bacteriocin PA166 was used as control.

Ten millimicrons of the cations (Na^+^, K^+^, Ga^2+^ and Mg^2+^ in bacteriocin PA166 were added to determine the activity of bacteriocin PA166. The untreated bacteriocin PA166 was used as control.

Bacteriocin PA166 was treated with different chemicals (ethanol, cysteine, EDTA, Triton X‐100, PMSF, β‐ME, DTT, Tween 20 and Tween 80) at 1% final concentration, and its activity was assessed. The untreated bacteriocin PA166 was used as control.

All samples were assayed for residual antibacterial activity using the agar diffusion assay.

### Antibacterial spectrum and MBCvalues of bacteriocin PA166

The antibacterial spectrum of bacteriocin PA166 was evaluated on the indicator strains (Table S1) using the agar well diffusion assay (Fredua‐Agyeman and Gaisford, 2019).

The minimum inhibitory concentration (MIC) of bacteriocin PA166 was defined as described previously (Qiao *et al*., 2020a, 2020b) with minor modifications. Briefly, 50 μl of twofold serial dilutions of bacteriocin PA166 were added to the cell suspension (the final concentration of 10^6^ CFU ml^−1^) in 96‐well plates. Then, the mixture was cultured at 37°C for 14 h. The MIC was defined by measuring OD at 600 nm using a microplate reader (Thermo Fisher Scientific Inc, Shanghai, China).

After MIC determination, 100 μl of the solution with no visible bacterial growth was inoculated into MH agar plates (containing 5% serum) at 37°C for 16 h. The minimum bactericidal concentration (MBC) was defined as the lowest antibiotic concentration that killed 99.9% of bacteria (Parvekar *et al*., 2020).

### Measurement of hemolytic activity

Bacteriocin PA166 was further purified according to a previous study (Dong *et al*., 2019a, 2019b). Healthy rabbit red blood cells were collected and washed three times using PBS and diluted to 1% with PBS. Then, 100 μl of blood cells were mixed with bacteriocin PA166 to give a final concentration of 1–256 μg ml^−1^, following which the mixtures were incubated at 37°C for 1 h. After centrifugation of the mixture (1000 *g*, 5 min), the supernatants were transferred to 96‐well plates. The release of haemoglobin was measured at OD = 570 nm.

### Cytotoxicity measurement

The effects of bacteriocin PA166 on the viability of Vero and NR8383 cell lines were measured using the CCK8 assay. The cells in Dulbecco's Modified Eagle Medium with 5% fetal bovine serum were incubated at 37°C in the presence of 5% CO_2_. Further, 50 μl of the cell suspension was mixed with a final concentration of bacteriocin PA166 (1–256 μg ml^−1^) in 96‐well plates. After 24 h, 10 μl of CCK‐8 solution was added and incubated for 4 h. The absorbance was read at 450 nm using an F4500 fluorescence spectrophotometer (Hitachi, Tokyo, Japan).

### Zebrafish embryo developmental toxicology assays

Zebrafish embryos were exposed to different concentrations of bacteriocin 48 h after fertilization, with 200 μg ml^−1^ sodium dehydroacetate as a positive control and embryo medium as a negative control according to a previous study (Song *et al*., 2020). The changes in zebrafish were observed under an inverted microscope after embryo culture at 28°C for 72 h.

### Mouse infection model

The therapeutic potential of bacteriocin PA166 was evaluated using a mouse infection model. The Kunming mice (6 weeks old, weighing ≈20 g) were randomly divided into four groups (10 mice per group). Three groups were inoculated with 100 μl 10 g^−1^ bacterial suspension containing 1.12 × 10^12^ CFU ml^−1^ of *P. multocida* 1 h after infection. One‐hour post‐infection, PBS (positive control), 4 mg kg^−1^ bacteriocin PA166 and 16 mg kg^−1^ bacteriocin PA166 were introduced intraperitoneally. The other group was inoculated with PBS as a negative control. Two days later, the lung tissues were harvested using bacterial counting, haematoxylin and eosin (H&E) staining, and microscopic observation.

### Time‐killing kinetics

The time‐killing kinetics assay was performed as described previously (Du *et al*., 2018) with minor modifications. The indicator strains were adjusted to an OD_600_ of 0.5 and exposed to MBC and bacteriocin PA166. The samples were collected every 4 h, 10‐fold serially diluted, and plated on MH agar plates (containing 5% serum). The viable cells were counted after 14 h.

### Release of ATP

The ATP released from the cells was measured using an ATP Test Kit. Briefly, the indicator bacteria were cultured to the mid‐log phase in MH, and the cells were collected by centrifugation (1000 *g* for 10 min). The cells were broken after adding 300 μl of ddH_2_O. Different reagents were added to 96‐well plates. The absorbance was read at 636 nm using a microplate reader (Thermo Fisher Scientific Inc).

### Release of ROS

ROS are involved in physiological and pathological processes such as cell growth, development, senescence and apoptosis. The level of ROS released from the cells was measured using a ROS assay kit. The ROS was tested based on the manufacturer's instruction. Briefly, *P. multocida* ATCC 43137 was grown overnight at 37°C, washed and resuspended in 10 mM of PBS to obtain an OD600 of 0.5. Ten μM DCFH‐DA was added, and the mixture was incubated at 37 °C for 30 min. After washing using 10 mM PBS three times, bacterial cells were added to a 96‐well plate and then 10 μl bacteriocin PA166 was added. After incubation at 37 °C for 30 min, the fluorescence was measured (excitation *λ* = 485 nm; emission *λ* = 525 nm) using an F4500 fluorescence spectrophotometer (Hitachi, Tokyo, Japan).

### DNA‐binding assay

The DNA‐binding assay was performed as previously described (Park *et al*., 2009). Briefly, 200 ng genomic DNA of *P. multocida* ATCC43137 was mixed with different concentrations of bacteriocin PA166 (0.5–256 μg ml^−1^) in a binding buffer for 1 h at room temperature. Then, the mixture was analysed using 1% agarose gel electrophoresis.

### Inner membrane permeability

The permeabilization of the inner membrane was determined by testing the intracellular β‐galactosidase activity. The inner membrane permeability was performed as previously described (Wang *et al*., 2019) with minor modifications. Briefly, the indicator bacteria were cultured in MH (containing 2% lactose and 5% serum) overnight. The cells were collected by centrifugation (1000 *g* for 5 min), washed three times and adjusted to an OD_600_ 0.05 using 5 mM HEPES endomembrane buffer (containing 1.5 mM ONPG and 20 mM glucose). The suspension was mixed with a final concentration of bacteriocin PA166 (1/4 MIC, 1/2 MIC, MIC, 2MIC and 4MIC) in 96‐well plates and incubated at 37°C. The OD_420_ values were measured every 5 min eight times.

### Outer membrane permeability assay

The fluorescent dye NPN was used to measure the outer membrane permeability. The indicator bacteria were washed thrice and adjusted to an OD_600_ 0.05 in 5 mM HEPES endomembrane buffer (containing 5 mM glucose). Then, 10 μM NPN was added and mixed with different concentrations of bacteriocin PA166 (1/4 MIC, 1/2 MIC, MIC, 2 MIC and 4 MIC). The fluorescence was defined (excitation *λ* = 350 nm, emission *λ* = 420 nm) using an F4500 fluorescence spectrophotometer (Hitachi, Tokyo, Japan). The outer membrane was converted into percent NPN uptake using the following equation:

$$\mathrm{NPN}\;\text{uptake}\;\left(\%\right)=\left(F_{\mathrm{obs}}-F_{0}\right)/\left(F_{100}-F_{0}\right)\times 100\%,$$

where *F*_obs_ is the observed fluorescence tested in the presence of the bacteriocin PA166, *F*_0_ is the initial fluorescence of NPN in the absence of bacteriocin and *F*_100_ is the fluorescence of NPN in the presence of 10 μg ml^−1^ polymyxin B.

### Cytoplasmic membrane depolarization assay

The indicator bacteria were washed thrice and adjusted to an OD_600_ 0.05 in 5 mM HEPES endomembrane buffer (containing 20 mM glucose and 100 mM KCl). Then, 0.4 μM DiSC3(5) was added and mixed with different concentrations of bacteriocin PA166 (1/4 MIC, 1/2 MIC, MIC, 2 MIC and 4 MIC). The fluorescence was defined (excitation *λ* = 622 nm, emission *λ* = 675 nm) using an F4500 fluorescence spectrophotometer (Hitachi, Tokyo, Japan).

### SEM and TEM characterization

For the scanning electron microscopy (SEM) and transmission electron microscopy (TEM) sample preparation, *P. multocida* ATCC43137 cells were incubated at 37°C for 4 h with bacteriocin PA166. The cells in the control groups were incubated with PBS, harvested and washed three times with PBS. Then, the cells were fixed with 2.5% glutaraldehyde. Finally, the samples were sent to Jilin University for further analysis.

### Statistical analysis

All experiments were repeated three times, and the data were analysed using GraphPad Prism 9.0. The data were presented as mean ± SD. *P* values of *<*0.05 indicated statistically significant differences. Statistical significance was indicated as *P < 0.05, **P < 0.01, ***P < 0.001 and ****P < 0.001.

## Conflict of interest

All authors have no conflicts of interest.

## Ethical approval

All animal procedures were performed according to the guidelines established by the Association for Assessment and Acceleration of Laboratory Animal Care International. Animal experiments were approved by the Ethics Committee of Jilin Agricultural University.

## Author contributions

Yu Wang participated in the conception and design of the study. Yu Wang, Yu Jia, Cheng‐guang He and Yue‐dong Liang conducted the antibacterial tests, outer‐membrane permeability, membrane depolarization, ATP and ROS assays and animal infection models. Yu Wang conducted the safety assessment. Yu Wang, Yi‐ming Wang, Ling‐cong Kong, Yun‐hang Gao, Xiao‐Ou Zhao, Emad Mohammed Elken and Hong‐xia Ma analysed the data. Yu Wang, M. Aman Haqmal and Inam Muhammad wrote the manuscript. All authors read and approved the manuscript.

## Supporting information


**Fig. S1** Purification of bacteriocin PA166. (A) Proteins were purified on a dextran gel chromatography column: the flow rate was 0.3 ml min^−1^. (B) First Q‐Sepharose column: proteins were eluted using five concentrations of NaCl (100, 200, 300, 400 and 500 mM). (C) Second Q‐Sepharose column: proteins were eluted using a linear gradient of NaCl (0–500 mM).

**Fig. S2** Mass spectra of 8 selected sequences. The mass spectrums are obtained by comparing the Uniprot database.

**Table S1** Bacterial strains and growth conditions.

**Appendix S1** The nucleotide sequence of the partial 16S rDNA gene sequence of *Pseudomonas* sp. strain 166.

## Data Availability

Source data supporting the findings of this study are included in the article.
