# Supplementary material for: Antibacterial activity and cytotoxicity of a novel bacteriocin isolated from Pseudomonas sp. strain 166
Source: Microb Biotechnol. 2022 Jul 18;15(9):2337–50. doi: 10.1111/1751-7915.14096 (PMC9437881; doi:10.1111/1751-7915.14096)

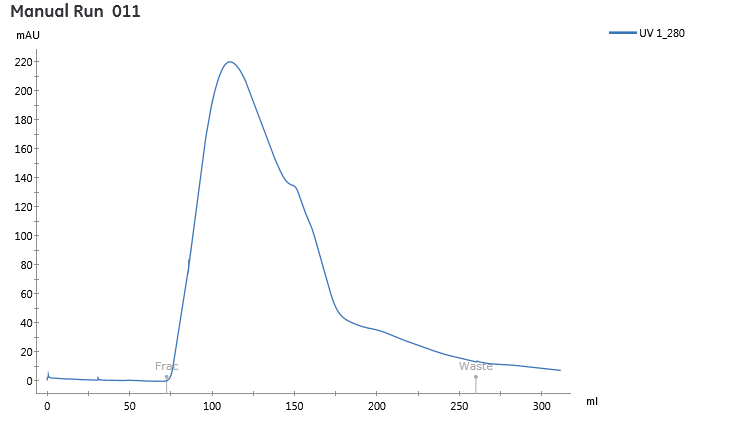

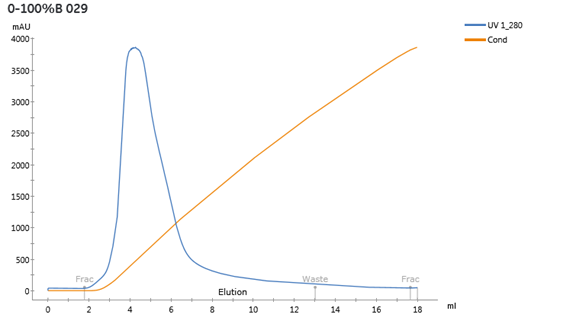

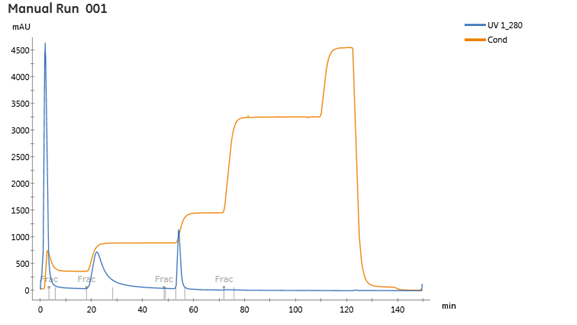


Figure S1. Purification of bacteriocin PA166 (a) Proteins were purified on a dextran gel chromatography column: the flow rate was 0.3ml/min (b) First Q- Sepharose column: proteins were eluted using five concentrations of NaCl (100, 200, 300,400 and 500 mM), (c) Second Q- Sepharose column: proteins were eluted using a linear gradient of NaCl (0~ 500 mM).

A

B

C


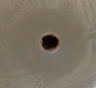

Supplement: Supplementary file 1 — Fig. S1 Purification of bacteriocin PA166. (A) Proteins were purified on a dextran gel chromatography column: the flow rate was 0.3 ml min−1. (B) First Q‐Sepharose column: proteins were eluted using five concentrations of NaCl (100, 200, 300, 400 and 500 mM). (C) Second Q‐Sepharose column: proteins were eluted using a linear gradient of NaCl (0–500 mM). [file MBT2-15-2337-s004.docx]
