# Supplementary material for: Antibacterial activity and cytotoxicity of a novel bacteriocin isolated from Pseudomonas sp. strain 166
Source: Microb Biotechnol. 2022 Jul 18;15(9):2337–50. doi: 10.1111/1751-7915.14096 (PMC9437881; doi:10.1111/1751-7915.14096)

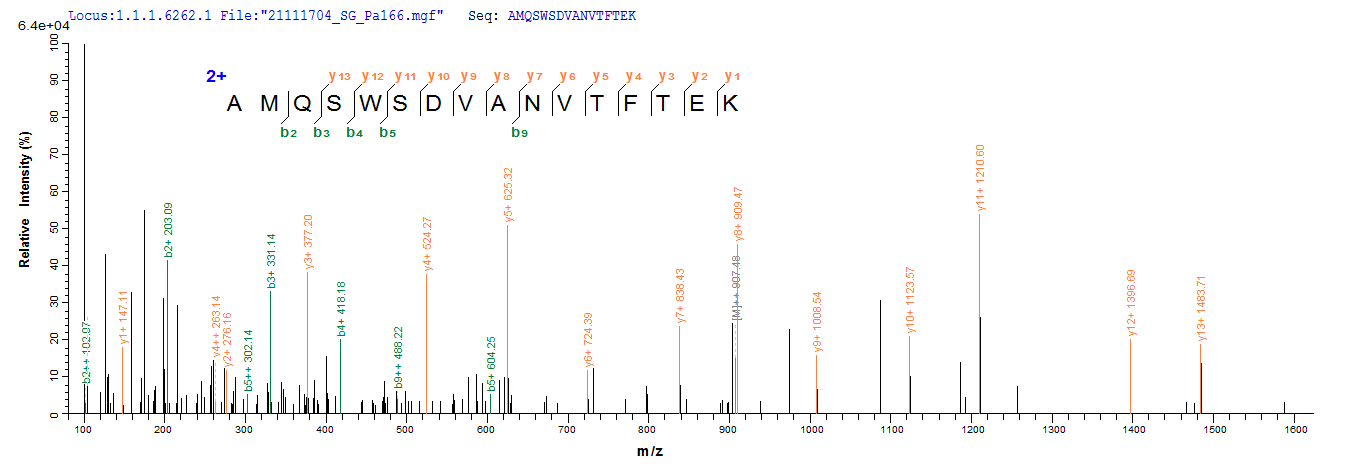

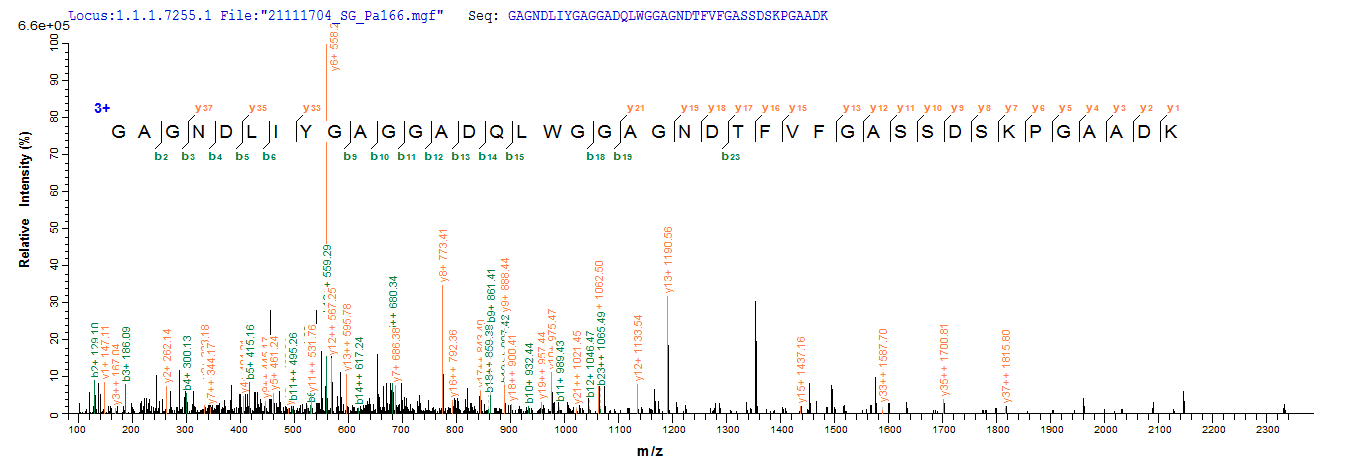

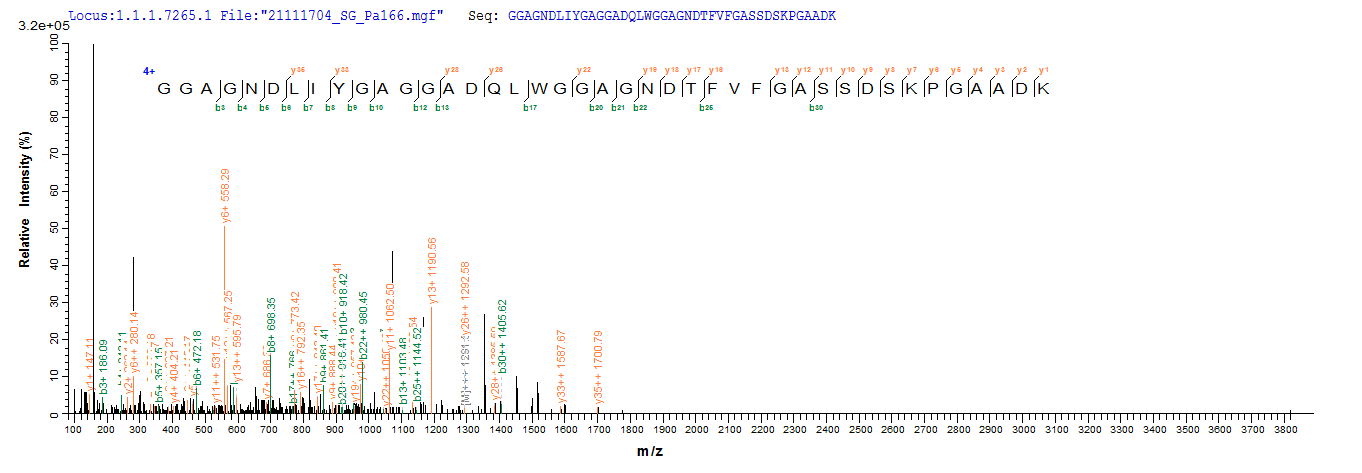

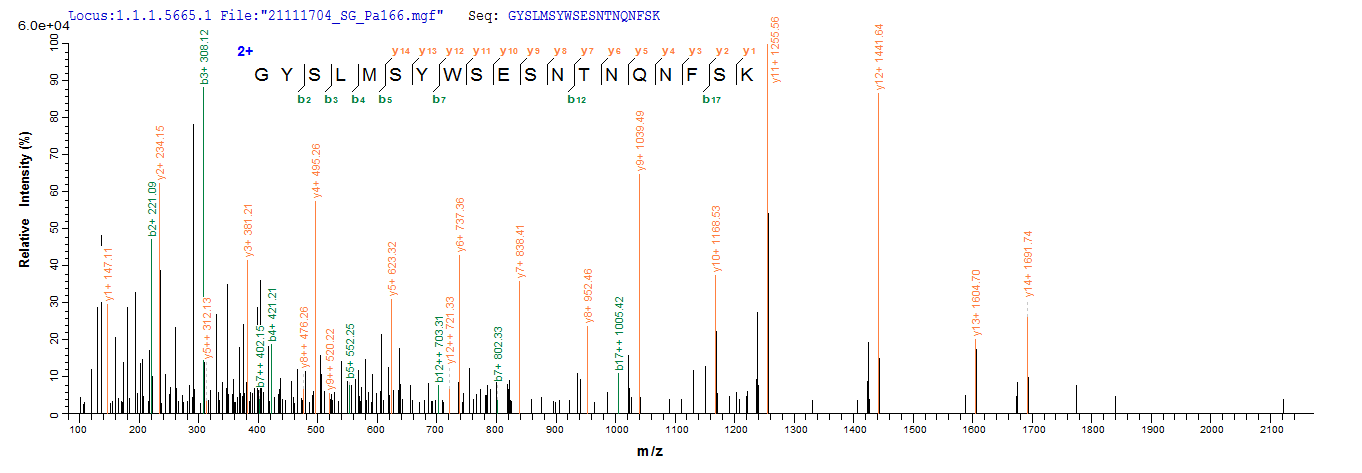


Fig. S2. Mass spectra of 8 selected sequences

The mass spectrums are obtained by comparing the Uniprot database


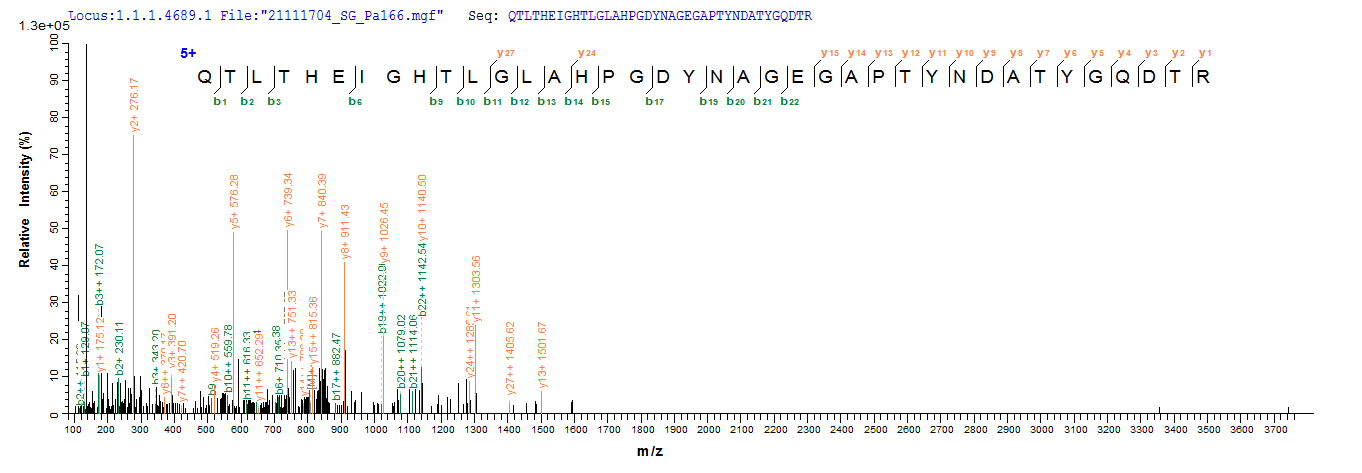

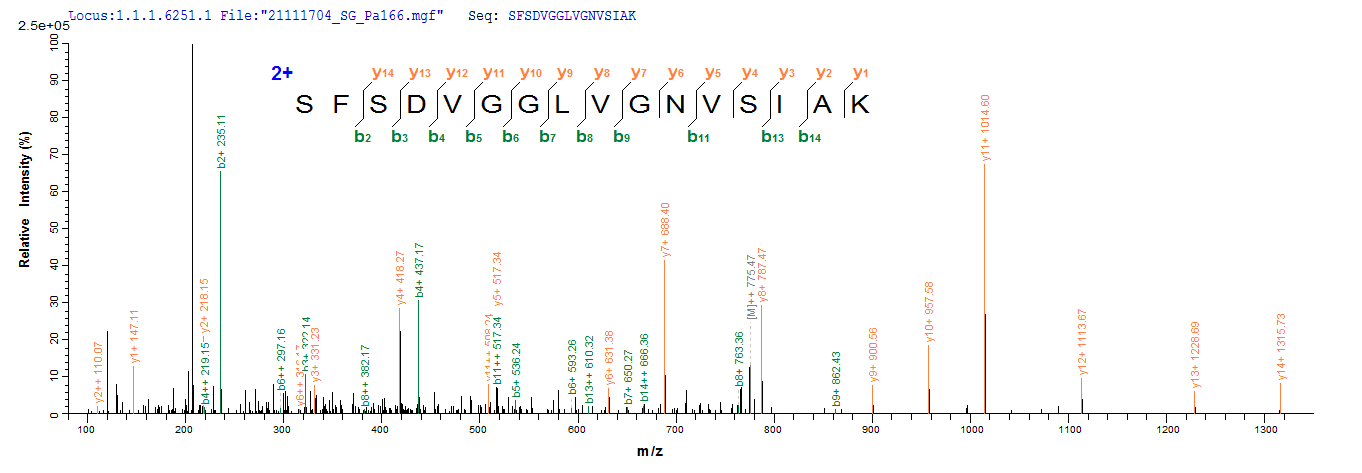

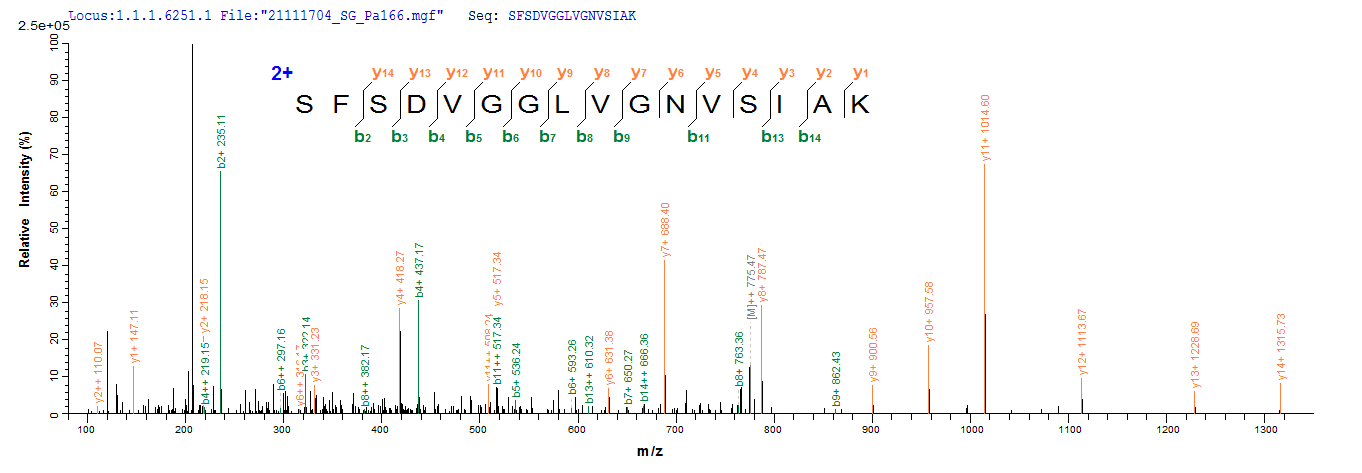

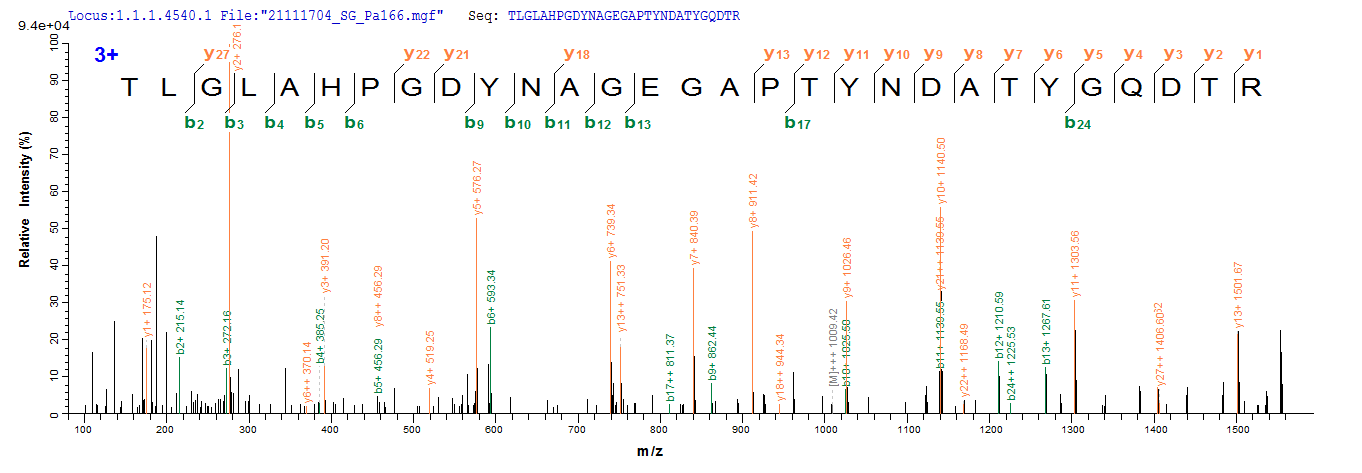

Supplement: Supplementary file 2 — Fig. S2 Mass spectra of 8 selected sequences. The mass spectrums are obtained by comparing the Uniprot database. [file MBT2-15-2337-s001.docx]
