# Supplementary material for: Antibacterial activity and cytotoxicity of a novel bacteriocin isolated from Pseudomonas sp. strain 166
Source: Microb Biotechnol. 2022 Jul 18;15(9):2337–50. doi: 10.1111/1751-7915.14096 (PMC9437881; doi:10.1111/1751-7915.14096)
Supplement: Supplementary file 3 — Table S1 Bacterial strains and growth conditions. [file MBT2-15-2337-s002.docx]

Table S1 Bacterial strains and growth conditions

| Strains | Medium | Incubation temp. (°C) |
| --- | --- | --- |
| *P. multocida* ATCC 43137 | MH (containing 5% serum) | 37 |
| *P. multocida* 32 | MH (containing 5% serum) | 37 |
| *P. multocida* 2 | MH (containing 5% serum) | 37 |
| *P. multocida* 6 | MH (containing 5% serum) | 37 |
| *P. multocida* 12 | MH (containing 5% serum) | 37 |
| *M. haemolytica* | MH (containing 5% serum) | 37 |
| *E. coli* B2 | MH | 37 |
| *E. coli* ATCC 25922 | MH | 37 |
| *S. enterica* subsp. enterica ATCC H9812 | MH | 37 |
| *E. fergusonii* | MH | 37 |
| *P. aeruginosa* | MH | 37 |
| *E. faecium* | MH (containing 5% serum) | 37 |
| *E. faecalis* | MH | 37 |
| *T.*pyogenes | MH (containing 5% serum) | 37 |
| MRSA | MH | 37 |
| *Candida albicans* | MH | 37 |
| *Phaffia rhodozyma* | MH | 37 |
