# Supplementary material for: Antibacterial activity and cytotoxicity of a novel bacteriocin isolated from Pseudomonas sp. strain 166
Source: Microb Biotechnol. 2022 Jul 18;15(9):2337–50. doi: 10.1111/1751-7915.14096 (PMC9437881; doi:10.1111/1751-7915.14096)
Supplement: Supplementary file 4 — Appendix S1 The nucleotide sequence of the partial 16S rDNA gene sequence of Pseudomonas sp. strain 166. [file MBT2-15-2337-s003.docx]

ctggccgata actgcaatta tccatccgat aattgtatcc gagttatttc ctttacaact

taaagggata tgacaatgtc ccgcggttct cttgcattga agggtcgcag caatgcgtaa

acccaggtag acaacttcct gcatgcctat gcgcgaggcg ggtacgaatt aatcggtggc

caacggtcct atagagtcga ccaggcggcg gagcagatcc tccgcgaaca ggcgtcttgg

cagcaggcac caggcgactc gatgctgacc ctgtggtatt cgttcctgac cacaccgaac

gacttcaaca atacgttgtg gccgtatgtc agcgatatct actcgctggg caagttcagc

gccttttccg cgcagcagca ggaacaggcc ttgttgtcgc tgcgttcctg ggcggacgtc

accaatatcc acttcgtcga cgccggccag ggcgatcagg gcgacctgac cttcggcaac

ttcagcagta gtgtcggcgg tgcggcgttc gccttcctgc cggatgtacc gaatgcggtc

aagttacaat cctaatacct gatcggcagc agctacagcg ccaacgtcaa accggccatc

ggcaactacg gacgccagac gctgacccac gagatcggcc ataccctgga cctgagccac

cccggcgact acaacgccgg cgaaggcgat ccgacctacg ccgacgctac ctacgccgag

gacacaagcg cctaatcggt gatgagctac tgagcagagc agcacagcga ctaccacttc

aatcacgcct attcctcgac accgtagctg gacgacatcg caaggatcca gacgctctac

ggaaccaacc tgaccacgcg caccggcgac acggtgtacg ggaacaactc caacattgag cgcgacttct acagcgccac ctcgtccagt tgcaagcttg tgtcctcggt gtggaacgcc ggcaccaacg acactgtgga cttctccagc ttcagccaga accagaagat caacctcaac gagaaggcgc tgtccgatgt cgtcgggtag acgtgcactg tgtcgatcgc tgccgggctc

aacgtcgaac acgtcatcgg cgactcgggt aacgaccggt tgatcgccaa cgacatgacc

agcgtgctca agtgcggcgc cgtcagcgac atcgtctgcg gcgacctcgg cgcatacgag

ctgtgtgttg gctagttagc cgacaggttc gtctacagcg atatcgctga gtcgtcaacg

gcagcgctgg atacccttcg cgactacgtt agcagccaag acttgatcga cctgtccagg

ctggacgcct tcgtcaacgg cgggctggtg ctccggtacg acgacgcctt cgccggcaag gccggccagg cgatcctgtc ctacgacgcg gcgagcaagg ccggcagcct ggcgatcgac ttcagcgggg atgcccatgc cgattccgcg atcaatctga tcggccagcc gacccaggcc gacatcgtcg tctgacgcgc tgatacgggc gcggttcggt gcggcggtac gaaccgcggc ccttcggccg gccgggcact gttcgacttg atagatgtct tccacaggag cgaagttgtg

Supplementary material 1. The nucleotide sequence of the partial 16S rDNA gene sequence of *Pseudomonas* sp. strain 166
